# Additional gastric resection after one anastomosis gastric bypass–are there benefits?

**DOI:** 10.3906/sag-2201-185

**Published:** 2022-04-07

**Authors:** Hacı Murat ÇAYCI, Umut Eren ERDOĞDU, Mehmet Akif TÜRKOĞLU, Ali TARDU, Ufuk ARSLAN, Gözde DOĞAN, Hasan ÇANTAY

**Affiliations:** 1 Department of General Surgery, Bursa Yüksek İhtisas Teaching and Research Hospital, University of Health Sciences, Bursa, Turkey; 2 Department of General Surgery, Faculty of Medicine, Gazi University Ankara, Turkey; 3 Department of General Surgery, Private Doruk Hospital, Bursa, Turkey; 4 Department of General Surgery, Faculty of Medicine, Kafkas University, Kars, Turkey

**Keywords:** Morbid obesity, one anastomosis gastric bypass, bariatric surgery, weight loss, mini gastric bypass, metabolic surgery

## Abstract

**Background/aim:**

Mini/one anastomosis gastric bypass (MGB-OAGB) is a bariatric surgery procedure that has proved effective for weight loss and the resolution of metabolic disorders. The present study evaluates the effect on postoperative outcomes of resecting the corpus and fundus as an addition to OAGB.

**Materials and methods:**

This retrospective study recorded and evaluated the data of 83 patients who underwent laparoscopic OAGB due to morbid obesity (Body Mass Index-BMI ≥ 40 kg/m^2^) in our clinic between January 2018 and January 2020. The patients were divided into two groups: the first group comprised patients undergoing standard OAGB (n = 49), while the second group included those undergoing OAGB plus (OAGB with additional corpus and fundus resection) (n = 34). The patient data recorded for comparison included demographic characteristics, comorbidities, preoperative and postoperative weight (at 6 and 12 months), body mass index (BMI), excess weight loss% (EWL%), excess BMI loss% (EBL%), and total body weight loss% (TBWL%), hemoglobin, fasting blood glucose (FBG), albumin and HbA1c levels.

**Results:**

There was no statistically significant difference between the two groups with regard to age, gender or comorbidities. The operating time, the number of cartridges used during the operation and the length of hospital stay were statistically higher in the OAGB plus group (p = 0.039, p < 0.001, p < 0.001, respectively). No statistically significant difference was seen between the groups regarding weight, BMI, EBL% and TBWL% preoperatively and at 6- and 12-months postsurgery. There was also no statistically significant difference in preoperative and postoperative (at months 6 and 12) levels of hemoglobin, FBG, albumin, and HbA1c between the two groups.

**Conclusion:**

The addition of resection of the gastric fundus and corpus to an OAGB has no impact on postoperative weight loss or metabolic outcomes.

## 1. Introduction

The incidence of bariatric surgery has increased in parallel with the prevalence of obesity and its associated comorbidities (e.g., hypertension, hyperlipidemia, and type 2 diabetes mellitus) [1]. Nowadays, sleeve gastrectomy (SG) followed by Roux-en-Y gastric bypass (RYGB) are the most commonly used bariatric surgical procedures and have long been considered as the optimal treatment modalities. However, recently, the mini/one anastomosis gastric bypass (OAGB-MGB) procedure, with its quick learning curve and operation time, low risk of internal herniation, and ability to provide intestinal stimulation and hypo-absorption, has begun to gain popularity [2,3]. By creating a long gastric pouch and, usually, a 200-cm jejunal omega loop, this procedure can produce effective weight loss and metabolic control (especially of type 2 diabetes mellitus) together with low morbidity and mortality rates [4–9].

Sleeve gastrectomy is a restrictive bariatric surgery method, providing effective weight loss through resection of the gastric fundus and corpus [10]. Ghrelin, a hormone associated with hunger, is released from the oxyntic cells that are especially abundant in the fundus; after resection, decreasing levels of this hormone cause reduction in the patient’s appetite [11]. On the other hand, although gastric bypass surgery creates a narrow gastric pouch, the results are malabsorptive rather than restrictive and metabolic-hormonal activity via the small bowel is its essential feature. Roushdy et al. have previously shown in a randomized controlled trial that the decrease in fasting ghrelin levels after sleeve gastrectomy is greater than that of OAGB [12]. There is limited information on supplementing a gastric bypass with fundus and corpus resections, as in sleeve gastrectomy, or on the effect of such additional procedures on postoperative outcomes. Therefore, the aim of the present study was to reveal whether there will be additional weight loss due to decreased ghrelin hormone secretion by adding fundus and corpus resection to the standard OAGB.

## 2. Subjects and methods

### 2.1. Study design

**Figure 1:**
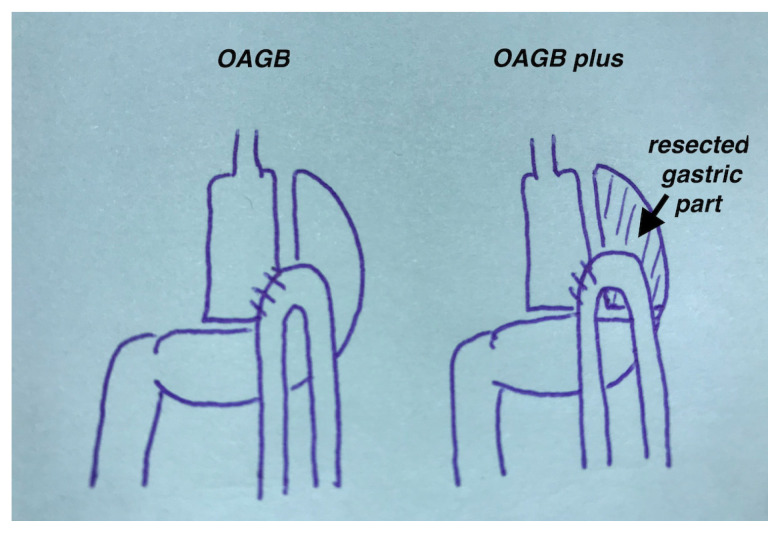
Schematic picture depicts forming a large and narrow gastric pouch with bypassing part of the small bowel. OAGB; one anastomosis gastric bypass, OAGB plus; one anastomosis gastric bypass with additional corpus and fundus resection

This retrospective study recorded and evaluated the data of 83 patients who underwent laparoscopic mini/one anastomosis gastric bypass (MGB-OAGB) to treat morbid obesity (body mass index-BMI ≥ 40 kg/m^2^) at the general surgery clinic of Health Sciences University Bursa Higher Specialization Training and Research Hospital between January 2018 and January 2020. The patients were divided consecutively into two groups: the first comprised patients undergoing standard OAGB (n = 49), while those undergoing OAGB plus (OAGB with additional corpus and fundus resection) made up the second group (n = 34) (Figure 1). Detailed information about the study procedure was provided to the patients prior to the operation, and their written informed consent for inclusion in the study was obtained. Ethical approval was not required for this study, given that the manuscript is retrospective research.

### 2.2. Sampling

The study included patients aged 18–65 years with a BMI ≥40 kg/m^2^, who experienced weight change postoperatively at months 6 and 12, and who underwent biochemical tests. Patients with a BMI ≤40 kg/m^2^, those having an alternative bariatric procedure, patients whose surgery needed revision and those whose preoperative endoscopic examination had revealed severe esophagitis, Barrett’s esophagus or gastroesophageal reflux disease, were excluded from the study. Thirteen patients who could not be reached during a one-year follow-up were excluded from the study. All patients were operated on at the same center and by the same surgical team. The morbidly obese patients were evaluated by a dietitian and a psychologist preoperatively and during postoperative follow-up. In the postoperative period, the patients were placed on a standard post-discharge diet, and were prescribed proton pump inhibitor (PPI) therapy for the first three months. Check-up visits were performed at 1, 3, 6, and 12 months. An upper gastrointestinal endoscopy was performed to obtain a biopsy from all patients preoperatively and one year postoperatively.

The patient data recorded for comparison included demographic characteristics and comorbidities; measurements taken preoperatively and postoperatively at 6 and 12 months of weight, body mass index (BMI), excess weight loss% (EWL%), excess BMI loss % (EBL%), and total body weight loss% (TBWL%), as well as hemoglobin (g/dL), fasting blood glucose (FBG) (mmol/L), and HbA1c (mmol/mol) levels.

### 2.3. Interventions

The first group received standard OAGB. Starting from the incisura angularis (the lesser curve), a gastric pouch was created using a 36-French bougie as a guide, and then a loop of small bowel was run 200 cm distal to the angle of Treitz. A 3-cm latero-lateral gastrojejunostomy anastomosis was performed in the posterior gastric wall using a 45-mm linear stapler. In the OAGB plus group, the stomach was divided completely longitudinally in a transverse fashion, starting from the incisura angularis. The proximal part of the stomach was divided up to the angle of HIS under the guidance of 36F gastric tube, as in sleeve gastrectomy, and the stomach was turned into a thin tube. As a result of division, the remaining fundus and corpus part of the stomach were removed by resection. The intestinal loop brought from the 200^th^ cm from the ligamentum of Treitz was anastomosed side-by-side to the posterior distal end of the tube stomach (Figure 1). In both groups, a methylene blue test was made to assess anastomotic integrity and the mesenteric defect (Petersen’s space) was not closed. The two groups received the same treatment during preoperative preparation and postoperative follow-up.

### 2.4. Statistical analysis

A Shapiro-Wilk test was used to analyze the normality of the preoperative and subsequent measurements. Measurements with normal distribution were reported as mean ± standard deviation, and those without normal distribution were reported as median (inter quartile range: IQR) values. The study groups were compared using the Independent Samples t-test and Mann-Whitney U-test for continuous variables, and Fisher’s exact test and Pearson’s chi-square test for categorical variables. All statistical analyses were carried out using the SPSS (IBM Corp. Released 2012. IBM SPSS Statistics for Windows, Version 21.0. Armonk, NY: IBM Corp.) software package, and a p value of <0.05 was considered statistically significant.

## 3. Results

The study included 49 patients in the OAGB group and 34 in the OAGB plus group. All surgeries were carried out laparoscopically. Patients were assessed for demographic characteristics, comorbidities, smoking, operating time, number of cartridges used, morbidity and length of hospital stay (Table 1). There was no statistically significant difference between the two groups in terms of age, gender or comorbidities (p > 0.05). Postoperative morbidity developed in two patients from each of the two groups (4.1% vs. 5.9%). In the OAGB group, one patient experienced intraluminal bleeding, and was conservatively treated with a blood transfusion, while a second patient was operated on after developing ileus due to a trocar site hernia. In the OAGB plus group, one patient needed medical treatment for tracheal edema, and another patient developed an anastomotic leak on postoperative day 2, which was sutured using relaparoscopy. No postoperative mortality was experienced in either group. The operating time, the number of cartridges used in the operation and the length of hospital stay were statistically higher in the OAGB plus group (p = 0.039, p < 0.001, p < 0.001, respectively). At the one-year postoperative mark, all patients underwent gastroscopy. Bile was detected in the stomach of two (4.08%) patients in the OAGB group and in one (2.94%) patient in the OAGB plus group while marginal ulcers were not identified endoscopically in either patient group. Additionally, no patients displayed symptoms of diarrhea, vitamin deficiency, biliary or gastroesophageal reflux disease (GERD).

A total of twenty (40.8%) patients in the OAGB group and 18 (52.9%) in the OAGB plus group had type 2 diabetes mellitus. At one-year postoperative, full remission was achieved in 16 (80%) patients in the OAGB group and 10 (56%) from the OAGB plus group. Diabetes medication was discontinued in four (20%) and eight (44%) of the patients in the OAGB and OAGB plus groups, respectively, and while their HbA1c levels had decreased, they were not within normal limits, and therefore could be considered as only in partial remission. Hypertension was observed in 20 (40.8%) and 13 (38.2%) patients in the OAGB and OAGB plus groups, respectively. At one year postoperative, hypertension medication was discontinued in 16 (80%) patients in the OAGB group and in 11 (84.6%) patients in the OAGB plus group.

**Figure 2:**
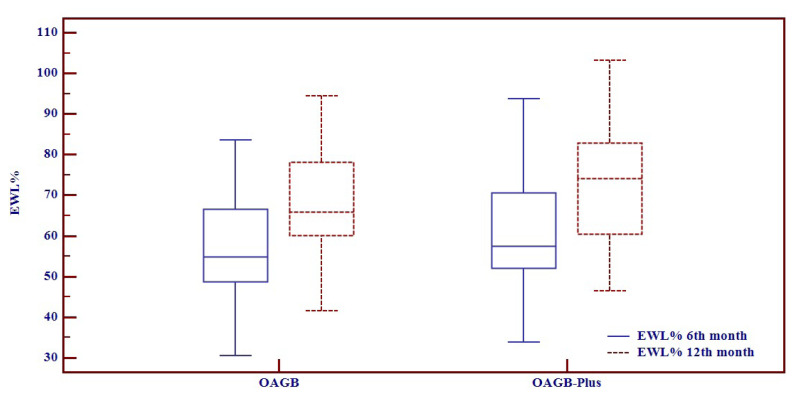
When the groups were compared using Mann Whitney U test, there was no statistical significance in terms of EWL % at 6th (p = 0.165) and 12th (p = 0.106) months following the operation, EWL%; Excess Weight Loss %

No statistically significant difference was established between changes in weight, BMI, EBL%, EWL% and TBWL% preoperatively and postoperatively (at 6 and 12 months) between the two groups (Table 2, Figure 2).

The patients were assessed preoperatively and postoperatively (at 6 and 12 months) for hemoglobin, FBG, albumin, B12 Vitamin and HbA1c levels (Table 3), and no statistically significant difference was noted between the groups for these parameters in the preoperative and postoperative periods (p > 0.05). The subgroup analyses for the diabetes mellitus patients also revealed no statistically significant difference between the groups (p > 0.05).

## 4. Discussion

The mini/one anastomosis gastric bypass (MGB-OAGB) procedure is a surgical method that can be learned more quickly than RYGB and is known for providing a malabsorptive effect [13]. In OAGB, a loop approximately 200 cm long is measured from the Treitz ligament, and an anastomosis with a width varying between 2–6 cm is made either manually or with a stapler [14]. There is, however, no standard length for the biliopancreatic limb [14]. In the present study, a 200-cm loop starting from the Treitz ligament, and a 3-cm wide anastomosis using an endoscopic stapler were standard procedures in both groups.

According to the literature, obese patients undergoing OAGB, record an EBL% of 72.9%–103.4%, EWL% of 65%–89% and TWL% of 36%–37.1% at the one-year postoperative check-up [5,6,9,14–19]. The EWL% reaches 48%–76.3% after six months [9,14,19]. Significant weight loss is achieved after one year (EWL% >50), and the weight loss process continues into the fifth year [20]. In the present study, the EBL% at 6 and 12 months postoperative, was 69.2% and 83% in the OAGB group, and 74.4% and 89.2% in the OAGB plus group; likewise, the EWL% was 57.04% and 68.48% in the OAGB group, and 61.03% and 73.3% in the OAGB plus group; and the TWL% was 30.5% and 36.7% in the OAGB group, compared to 31.7% and 38.3% in the OAGB plus group. Thus, both groups of patients were seen to lose weight effectively after OAGB, and the overall outcomes were consistent with previous studies. While the OAGB plus group recorded slightly better results, there was no statistically significant difference, although a clearer picture could possibly be achieved in a study involving a larger number of patients and a longer follow-up.

OAGB is reported to be an easier procedure for surgeons to master compared to LRYGB [5]. The operating time varies from between 27 to 210 min, in parallel with this learning curve; the quicker procedure results from performing only a single anastomosis in addition to no closure of the Peterson’s space [14]. In the present study, the operating times were 113.8 min and 137.2 min and the number of cartridges used was 6.22 and 7.76 in the OAGB and OAGB plus groups, respectively. Consistent with our previous experience, our operating times were not particularly short and, clearly, the additional resection during the OAGB plus procedure prolonged operating time and increased the number of cartridges used (p > 0.05).

Following OAGB, the expected perioperative morbidity rate is in the 2.7%–9% range with a mortality rate of 0%–0.5% [14,16]. The most common cause of morbidity is bleeding (0.2%–2.5%) and leakage from the stapler line or anastomosis (0%–5%) [9,12,14,16,17,21,22]. Less often, a trocar site or internal hernia, gastric stasis and stomal stenosis may occur [9,16]. In the present study, both groups had similar rates of morbidity (5.9% vs. 4.1%), and, in line with previous studies, bleeding that could be treated conservatively, as well as leakage that required reoperation occurred in our patients. There was no incidence of mortality in this series of patients.

According to the literature, the mean length of hospital stay after OAGB varies from 1–5.4 days [14,15,18]. In the present study, while the standard OAGB patient group was hospitalized for a mean of 5.3 days, the mean length in the OAGB plus group was 6.98 days, giving a statistically longer stay for the OAGB plus group (<0.001). Meanwhile, our overall longer hospitalization times are consistent with our previous experience, and most likely stem from our free healthcare service.

After OAGB, patients are known to achieve effective weight loss and improvement in metabolic disease (especially in type 2 diabetes mellitus) [23,24]. The mean rates of remission for patients with type 2 diabetes mellitus (T2DM) and hypertension, following OAGB, are 83.7% and 66.9%, respectively [14]. HbA1c levels are reported to plateau after OAGB, with a mean level of 5.2 mmol/mol after two years [20]. In the present study, FBG levels at the 6^th^ and 12^th^ months were 91 and 86 mmol/L, respectively, in the OAGB group, compared to 85 and 84 mmol/L, respectively, in the OAGB plus group. HbA1c at month 6 and month 12, were 5.3 and 5.4 mmol/mol, respectively in the OAGB group, and 5.44 and 5.38 mmol/mol, respectively in the OAGB plus group. At 12 months after surgery, full remission was achieved in 16 (80%) and 10 (56%) patients respectively, in the OAGB and OAGB plus groups. Diabetes medication was discontinued in four (20%) OAGB and eight (44%) OAGB plus patients, and their HbA1c levels were lowered; however, as the levels were still not within normal limits, these cases were only considered as partial remission. Regarding full and partial diabetes remission, the fundus and corpus resection added to OAGB did not produce any statistically significant difference. At one year postoperative, 80% and 84.7% of patients in the OAGB and OAGB plus groups, respectively, showed remission of hypertension. The high remission rates for hypertension and the difference in remission rates for patients with diabetes are believed to result from the low number of patients in each group.

The iron deficiency, anemia, has been reported to occur in 0.64%–15% of patients after OAGB, sometimes continuing into the fifth postop year; and is believed to develop due to the malabsorptive nature of the OAGB method [5,14]. In the present study, the hemoglobin levels at months 6 and 12 were 12.9 and 12.8 in the OAGB group, and 13.07 and 12.93 in the OAGB plus group, respectively, indicating no significant development of anemia in general. One possible reason for this is thought to be the routine use of multivitamin supplements containing B12 and iron throughout the first postoperative year.

Malnutrition may be seen in 0%–3.8% of patients after OAGB [14]. There is a lack of consensus on the optimum length of bowel (150–300 cm) for an effective anastomosis, although a loop longer than 200 cm is not recommended, as it would cause malabsorption and protein-calorie malnutrition. Also, proportioning should be made considering the total small bowel length, if possible [8,14]. In the present study, both groups had similar levels of albumin and no malnutrition, a probable result of the close support of a dietitian and the use of a standard 200-cm biliopancreatic limb length.

The bile reflux in the gastric pouch may occur in 0%–0.7% of patients, being mostly temporary and rarely symptomatic [13,17,23,25,26]. In the present study, a gastroscopic examination of the patients one year after surgery identified bile in the gastric pouch in 3.1% of patients [OAGB: n = 2 (4.08%); OAGB plus: n = 1 (2.94%)], although they were asymptomatic. Furthermore, neither group of patients presented with marginal ulcers, biliary or GERD symptoms.

### 4.1. Limitations

Our study is limited by its single-center design, its lack of quality of life and Ghrelin analyses, the relatively low number of patients in each group and the short one-year follow-up duration. In addition, the study was planned retrospectively to avoid possible bias in the evaluation of the data.

## 5. Conclusion

The number of bariatric surgical interventions performed for the treatment of morbid obesity are increasing, and methods are being developed aimed at achieving better outcomes. It was found that adding gastric fundus and corpus resections to OAGB, similar to sleeve gastrectomy procedures, had no impact on postoperative weight loss or metabolic resolution outcomes. On the contrary, additional gastric resections were found to increase cost and operating times. We believe that further studies involving a larger number of patients and a prolonged follow-up period are required to expand our understanding of how gastric resections may support OAGB surgery.

## Figures and Tables

**Table 1:** Demographic data, comorbidities and perioperative data of patients in the two groups.

	OAGB (n = 49)	OAGB-Plus (n = 34)	P
**Age**	44.1 ± 10.5	44.03 ± 9.6	0.960^a^
**Gender F/M**	42/7	29/5	>0.99^b^
**Comorbidities (+)**	25 (51%)	22 (64.7%)	0.216^c^
**T2DM (+)**	20 (40.8%)	18 (52.9%)	0.276^c^
**Smoking (+)**	13 (26.5%)	8 (23.5%)	0.757^c^
**Morbidity**	2 (4.1%)	2 (5.9%)	>0.99^b^
**Operating time (min)**	113.8 ± 21.8	137.2 ± 54.8	0.039^d^
**Number of Cartridges**	6.2 ± 0.8	7.7 ± 1.1	<0.001^d^
**Length of hospital stay (days)**	5.3 ± 1.5	6.9 ± 3.1	<0.001^d^

One anastomosis gastric bypass: OAGB. Data presented as mean ± SD. deviation, median (Interquartile Range: IQR) and n (%), M: Male, F: Female, T2DM: Type 2 Diabetes Mellitus

a Independent Samples t-test,

b Fisher’s exact test,

c Pearson chi-square test,

d Mann Whitney U-Test

**Table 2:** Preoperative and postoperative (6 and 12 months) weight and change rates.

		OAGB (n = 49)	OAGB-Plus (n = 34)	P

**Weight (kg)**	Preoperative	119.6 ± 15.2	118.5 ± 14.2	0.734^a^
	6^th^ month	81 (16.5)	80 (18.5)	0.236^d^
	12^th^ month	74 (15.5)	72.5 (14.7)	0.081^d^

**BMI (kg/m** **^2^** **)**	Preoperative	44.3 (8.5)	45.1 (6.7)	0.229^d^
	6^th^ month	31.9 ± 4.3	30.4 ± 4.2	0.140^a^
	12^th^ month	28.9 ± 3.9	27.4 ± 3.9	0.270^a^

**EWL%**	6^th^ month	57.0 ± 11.7	61.0 ± 14.2	0.165^a^
	12^th^ month	68.4 ± 11.8	73.3 ± 15.3	0.106^a^

**EBL%**	6^th^ month	69.2 ± 15.0	74.4 ± 17.7	0.154^a^
	12^th^ month	83.0 ± 15.6	89.2 ± 18.7	0.105^a^

**TWL%**	6^th^ month	30.5 ± 5.1	31.7 ± 6.1	0.323^a^
	12^th^ month	36.7 ± 5.7	38.3 ± 7.3	0.285^a^

One anastomosis gastric bypass: OAGB. Data were presented as mean ± SD. deviation and median (Interquartile Range: IQR)

BMI: Body mass index, EWL: Excess weight loss, EBL: Excess body mass index loss,

a Independent Samples t-test,

d Mann-Whitney U-Test

**Table 3:** Preoperative and postoperative biochemical changes.

		OAGB (n = 49)	OAGB-Plus (n = 34)	p-value

**Hemoglobin (g/dL)**	Preoperative	13.3 ± 1.6	13.5 ± 1.6	0.560^a^
	6^th^ month	12.9 ± 1.5	13.0 ± 1.1	0.803^a^
	12^th^ month	12.8 ± 1.2	12.9 ± 1.3	0.728^a^

**FBG (mmol/L)**	Preoperative	104 (80)	105 (142.2)	0.431^d^
	6^th^ month	91 (16)	85.5 (16.50)	0.138^d^
	12^th^ month	86 (13.5)	84.5 (20.5)	0.650^d^

**Albumin (g/L)**	Preoperative	4.4 ± 0.4	4.5 ± 0.2	0.440^a^
	6^th^ month	4.2 (0.5)	4.3 (0.4)	0.258^d^
	12^th^ month	4.2 (0.6)	4.3 (0.4)	0.514^d^

**HbA1c (mmol/mol)**	Preoperative	6.2 (2.6)	6.3 (3.9)	0.203^d^
	6^th^ month	5.3 (0.6)	5.4 (1.1)	0.364^d^
	12^th^ month	5.3 (0.4)	5.3 (0.7)	0.060^d^

One anastomosis gastric bypass: OAGB, FBG; Fasting Blood Glucose. Data were presented as mean ± SD. deviation and median (Interquartile Range: IQR)

a Independent Samples t-test,

d Mann-Whitney U-Test
